# Clinical efficacy study on the combined treatment of cancer-related depression with traditional Chinese acupuncture-related therapies and drugs

**DOI:** 10.3389/fpsyt.2025.1717290

**Published:** 2026-01-05

**Authors:** Ying Zhou, Feiqing Wang, Bo Yang, Xu Yang, Xiaoxu Chen, Bingbing Li, Yanqing Liu, Zhenhua Liu, Yang Liu, Dongxin Tang, Yanju Li

**Affiliations:** 1 Clinical Medical Research Center, The First Affiliated Hospital of Guizhou University of Traditional Chinese Medicine, Guiyang, Guizhou, China; 2 Academy of Medical Engineering and Translational Medicine, Tianjin University, Tianjin, China; 3 Department of Hepatobiliary Surgery, Guizhou Provincial People’s Hospital, Guiyang, Guizhou, China; 4 Department of Hematology Oncology, Affiliated Hospital of Guizhou Medical University, Guiyang, Guizhou, China

**Keywords:** acupoint application, acupuncture, acupuncture-related therapies, auricular acupressure, auricular point, cancer-related depression

## Abstract

**Background:**

The incidence of cancer-related depression (CRD) is constantly increasing. Some clinical practice guidelines have pointed out that traditional Chinese acupuncture-related therapies can serve as an effective supplementary therapy to drug treatment for enhancing the therapeutic outcome. In this study, we systematically evaluated the therapeutic efficacy and clinical significance of different acupuncture-related therapies in the treatment of CRD.

**Methods:**

Three English databases (PubMed, Cochrane, and Web of Science) and three Chinese databases (VIP Chinese Science and Technology Journal Database, China National Knowledge Infrastructure, and Wanfang Database) were searched up to October 2024. The primary outcome measures were the total effective rate, the Self-Rating Depression Scale (SDS), and the Hamilton Depression Scale (HAM-D).

**Results:**

This study included 30 RCTs, encompassing 2,886 patients. Among them, 1,446 were in the control group and 1,440 in the experimental group. The primary outcome indicators revealed that compared with drug therapy alone, drug therapy combined with Traditional Chinese acupuncture-related therapies demonstrated significant advantages in terms of efficacy (OR = 3.74, 95% CI 2.83, 4.96; *P* < 0.00001), SDS score (MD = -7.70, 95% CI -9.54, -5.85; *P* < 0.00001), and HAM-D score (MD = -3.77, 95% CI -4.98, -2.56; *P* < 0.00001). The results of subgroup analysis showed that after the combination of acupuncture-related therapies and drug treatments, The combined use of auricular points and drug therapy achieved the highest overall effectiveness rate (96.3%), followed by the combination of acupuncture-related therapies (91.8%), acupoint pressing and application (91.5%), and acupuncture (88.3%).

**Conclusion:**

Traditional Chinese acupuncture-related therapies, especially auricular acupressure therapy, can assist drug treatment in relieving CRD. This research facilitates the development of complementary medicine, and is conducive to reducing drug resistance and enhancing the quality of life of patients.

**Systematic review registration:**

https://www.crd.york.ac.uk/prospero/, identifier CRD420251242774.

## 1 Introduction

Cancer-related depression (CRD) is a common psychological disorder of depressive symptoms among cancer patients. These symptoms may result from the combined effects of the direct impact of the disease, side effects of treatment, augmented psychological stress, and the deterioration of the quality of life (1). The incidence of CRD can be as high as 58%, a figure that is three to four times that of the general population (2). CRD not only significantly undermines therapeutic efficacy but also severely reduces patients’ quality of life, influencing the ultimate prognosis of the disease. The prevalence and risk of this psychological state means that clinicians have begun to incorporate CRD treatment into comprehensive treatment regimens (3). At present, treatment for CRD mainly consists of drug therapy based on Western medicine and psychological intervention (4). However, these methods still have certain shortcomings including adverse reactions, which may impact the tolerance and treatment compliance of patients (5).

In this context, traditional Chinese medicine (TCM) techniques have shown certain advantages. Some research indicates that traditional Chinese medical techniques might alleviate depressive symptoms through regulating the corticostriatal reward/motivation circuit and the calcium/calmodulin-dependent protein kinase (CaMK) signaling pathway (6). This finding offers a novel therapeutic perspective for cancer patients, enhancing their quality of life and reducing the risk of accidental death caused by depression. Hence, researchers recommend implementing comprehensive intervention measures, including non-pharmacological treatments, for patients with CRD (7). Traditional Chinese acupuncture-related therapies, such as acupuncture, moxibustion, acupoint application with patches, acupoint pressure, and auricular acupoint pressure, have been extensively applied worldwide and have gradually gained recognition from the international community (8, 9). According to the National Administration of Traditional Chinese Medicine of China, the influence of TCM has extended to 196 countries and regions, and the number of people receiving TCM globally is rising each year (10). The development and dissemination of TCM have a solid foundation, which is manifested as follows: acupuncture has been incorporated into the medical insurance systems of 18 countries, and over 30 countries and regions have established TCM colleges (11).

**Figure 1:**
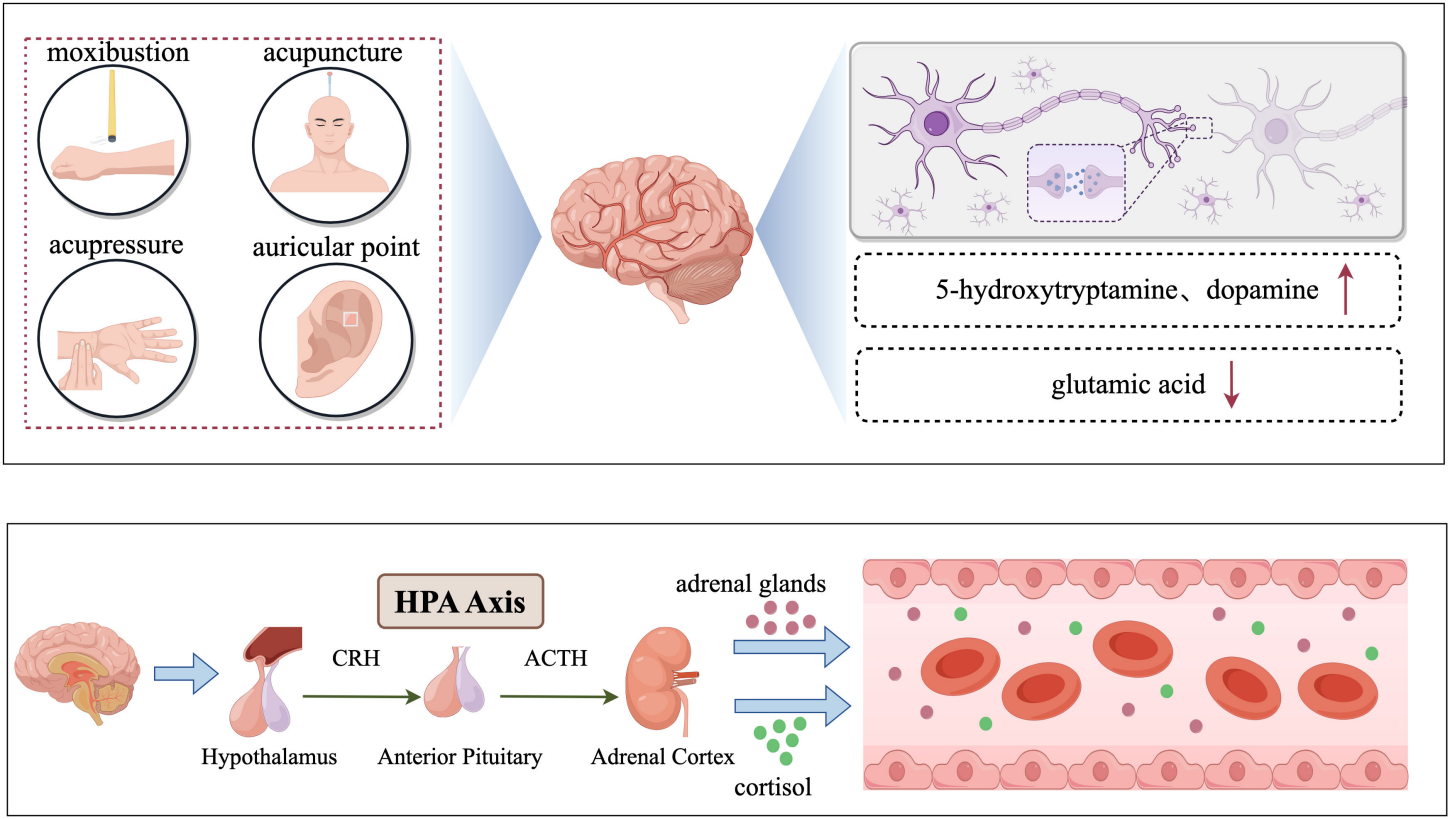
Traditional Chinese acupuncture-related therapies for treating cancer-related depression.

This study aimed to investigate the clinical efficacy of different acupuncture therapies in the treatment of CRD, and to examine its effectiveness and safety. We anticipate that this study will allow a more comprehensive and effective treatment alternative to be offered to cancer patients. As a non-pharmacological treatment, the advantage of traditional Chinese acupuncture-related therapies lies in its ability to specifically regulate the physiological functions of the human body, thereby alleviating depressive symptoms without incurring the adverse reactions present in traditional drug therapies. The flow chart of the study is shown in **Figure 1**.

## 2 Methods

This research was carried out in accordance with the Preferred Reporting Items for Systematic Reviews and Meta-Analyses (PRISMA) reporting guidelines (12). This research plan can be obtained from PROSPERO (CRD420251242774).

### 2.1 Literature search strategy

#### 2.1.1 Eligibility criteria

Literature type: Randomized controlled trials (RCTs) on traditional Chinese acupuncture-related therapies for acupuncture points (acupuncture, moxibustion, acupoint application, auricular point, auricular acupressure) for CRD. Patients: patients with a definite diagnosis of CRD. There are no requirements for tumor type, gender, or age of the patients, but comparability is necessary. Intervention measures: The control group received conventional drug treatment for cancer-related depression, without combining traditional Chinese acupuncture therapy or other non-drug intervention measures; the experimental group, in addition to the above-mentioned conventional drug treatment, added traditional Chinese acupuncture-related therapies (either as a single therapy or in combination of 2–3 therapies). Outcome indicators: This study divided the outcome indicators into primary outcome indicators and secondary outcome indicators. The main outcome indicators are the total effective rate, the score of the Self-Rating Depression Scale (SDS), and the score of the Hamilton Depression Scale (HAM-D). The secondary outcome indicators were the Self-rating Anxiety Scale (SAS), Pittsburgh Sleep Quality Index (PSQI), Numeric Rating Scales (NRS) for cancer pain and quality of life (QOL). The quality of life questionnaire-core 30 scale was divided into the main symptom domain score, single domain score and functional domain score.

#### 2.1.2 Exclusion criteria

Literature type: non-randomized controlled trials such as reviews, case reports, conference papers, commentaries, and solicited contributions. Patients: conventional treatments that incorporate oral Chinese medicine, without detailed descriptions of the names, types, or operational methods of acupuncture-related treatments; or in combination with other unspecified treatment measures; or cancer patients with a previous history of depression. Intervention measures: literature that does not clearly define or include the outcome indicators required for this study, or literature with inaccurate data that affect the judgment of outcome indicators. Outcome indicators: literature for which the original text cannot be obtained, and duplicate literature included in different databases.

### 2.2 Information sources and search strategy

RCTs related to CRD treatment by Traditional Chinese acupuncture-related therapies published from the establishment of the database to September 31, 2025 were retrieved successively from six English and Chinese databases including PubMed, Cochrane, Web of Science, VIP, Wanfang Data, and CNKI. The Chinese search terms “tumor”, “cancer”, “depression”, “acupuncture”, “moxibustion”, “acupoint application”, “acupoint compression”, and “auricular points” were used, and the English search terms “cancer”, “tumour”, “depression”, “acupuncture”, “moxibustion”, “acupressure”, and “auricular acupuncture” were used. A cross-search method of free words and subject terms was adopted (**Appendix 1**).

### 2.3 Data extraction and quality assessment

After removing duplicates, two researchers re-screened the identified studies according to the inclusion criteria. From the included literature, they extracted the following data: the name of the first author, publication year, the gender and age of the subjects, the sizes of the control and experimental groups, the intervention measures, the intervention duration, the outcome indicators, etc. Two researchers verified and entered the data, and differences of opinion were resolved through discussion with a third party. Assessment was carried out using the risk of bias tools of the Cochrane Collaboration (13) and classified into three risk groups: “low risk”, “high risk”, and “unknown risk”, as well as three quality grades: A, B and C. At the same time, the funnel graph analysis was used to evaluate the publication bias of the articles.

### 2.4 Statistical analysis

Statistical analysis was performed using RevMan 5.4 software. The effect indicator for count data was the odds ratio (OR), and for measurement data was mean difference (MD) or standardized mean difference (SMD). The 95% confidence interval (CI) was calculated. A Chi-square test was employed to analyze the statistical heterogeneity. If the test results indicated that I^2^ < 50%, the statistical heterogeneity among studies was considered to be relatively small, and the fixed effect model was chosen. If the test results revealed that I^2^ ≥ 50%, the statistical heterogeneity among studies was significant, and the random-effects model was adopted. A *P* value < 0.05 suggested that the test result was statistically significant.

## 3 Results

### 3.1 Study selection

**Figure 2:**
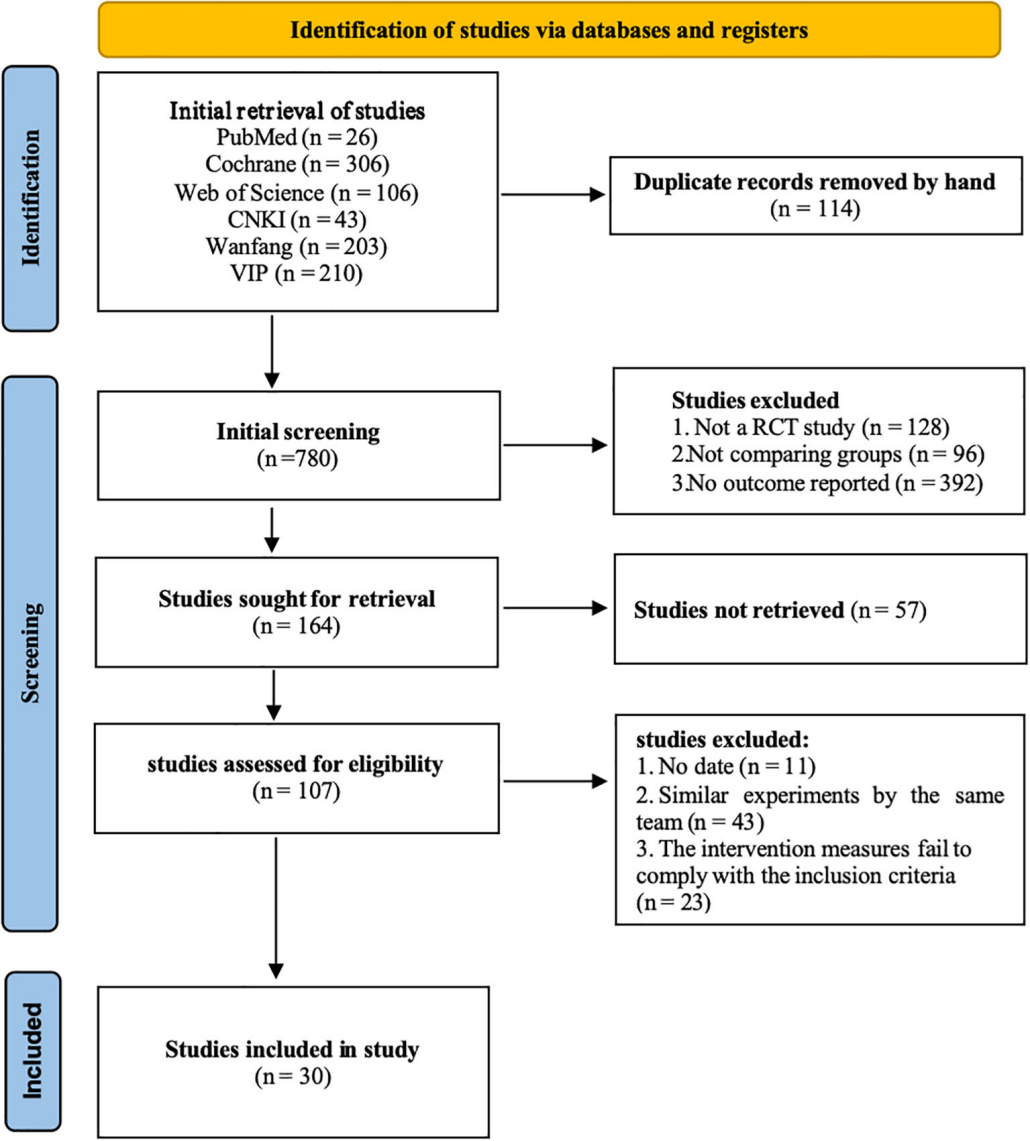
PRISMA flow chart for literature screening.

A total of 894 relevant studies were retrieved, among which 456 were in Chinese and 438 were in English. After manually eliminating 114 duplicates, 780 were incorporated. After reading the titles and abstracts and excluding those not meeting the inclusion criteria, 164 were eligible. After excluding those for which the original text could not be obtained even after contacting the authors and relevant institutions, 107 were included. After reading the full texts and excluding similar studies conducted by the same research team and those with intervention measures that did not conform to the inclusion criteria, 30 were ultimately eligible (**Figure 2**).

### 3.2 Study characteristics

The 2886 patients included were all clearly diagnosed with cancer-related depression, with an age range of 18–80 years and a balanced gender distribution. The experimental group received traditional Chinese acupuncture-related therapy combined with Western medicine treatment, including acupuncture (10 studies), moxibustion (5 studies), acupoint pressing (1 study), acupoint application (3 studies), ear acupressure (6 studies), and 2–3 combined therapies (5 studies), with intervention periods ranging from 1 to 12 weeks. The control group only received conventional Western medicine treatment. The main outcome indicators were total effective rate, SDS score, HAM-D score; secondary outcome indicators included SAS score, PSQI score, NRS pain score, and QOL core 30 scale score, etc. The 30 included studies were all randomized controlled trials (RCTs), and the publication time range was from 2010 to 2025 (**Table 1**).

**Table 1:** Characteristics of the included randomized controlled trials for CRD.

Name	Sample size (cases)		Age (years)		Interventions		Treatment cycle	Acupoints
	Exp	Con	Exp	Con	Exp	Con		
Pei Y 2010 (14)	31	36	51.76 ± 10.21	48.34 ± 8.79	Citalopram Hydrobromide	Acupuncture	6 weekss	Feishu, Xinshu, Geshu, Pishu, et al.
Feng Y 2011 (15)	40	40	63.80 ± 5.47	63.6 ± 4.26	Fluoxetine Hydrochloride	Acupuncture	4 weekss	Yintang, Baihui, Neiguan, Shenmen
Xia Q 2017 (16)	23	23	NA	NA	Fluoxetine Hydrochloride	Acupuncture	4 weeks	Scalp Acupuncture (Middle Line of Vertex (MS5), et al.)
Deng XY 2019 (17)	30	30	53 ± 9	49 ± 11	Sertraline Hydrochloride	Acupuncture	4 weeks	Hegu, Taichong, Neiguan, Shenmen, et al.
Liu YP 2019 (18)	40	40	63 ± 13	63 ± 12	Sertraline Hydrochloride	Acupuncture	4 weeks	Taichong, Hegu, Baihui, Yintang
Lian JL 2020 (19)	60	60	62.25 ± 5.43	62.25 ± 5.43	Sertraline Hydrochloride	Acupuncture	6 weeks	Baihui, Shenmen, Taichong
Chen Z 2021 (20)	54	54	54 ± 5	54 ± 4	Oxycodone Hydrochloride	Acupuncture	1 week	Mingmen, Zhishi, Shenshu, Zusanli, et al.
Li JX 2022 (21)	42	43	56.62 ± 5.75	55.93 ± 5.11	Flupentixol	Acupuncture	4 weeks	Danzhong, Neiguan, Hegu, Taichong
Zheng MY 2024 (22)	31	32	54.80 ± 6.12	56.46 ± 5.28	Flupentixol	Acupuncture	12 weeks	Shenmen, Sanyinjiao, Liangqiu, Zusanli, et al.
Zeng XL 2015 (23)	29	28	60.28± 10.22	61.71 ± 11.58	Sertraline Hydrochloride	Acupuncture	4 weeks	Neiguan, Shenmen, Laogong, et al.
Qian ZP 2021 (24)	22	22	36.33 ± 2.65	36.12 ± 2.52	Routine Treatmentt	Moxibustion	4 weeks	Zusanli
Zong TT 2023 (25)	35	35	60.9 ± 8.66	57.7 ± 9.45	Alprazolam	Moxibustion	2 weeks	Heat-sensitive Moxibustion: Naiguan, Yanglingquan
Chen YH 2013 (26)	30	30	NA	NA	Analgesics	Moxibustion	2 weeks	Zusanli, Xuehai, Sanyinjiao, Zhongwan
Xia WM 2018 (27)	37	37	47.6 ± 8.2	46.2 ± 7.5	Routine Treatmentt	Moxibustion	1 week	Baihui
Sun YH 2024 (28)	27	27	58.76± 12.03	60.33 ± 12.94	Routine Treatmentt	Moxibustion	6 weeks	Fuyang Moxibustion
Wang YJ 2017 (29)	35	35	53.61 ± 7.09	52.41 ± 7.32	Escitalopram Oxalate	Acupressure	10 weeks	Baihui, Shangxing, Shenmai, Taichong, et al.
Zhang GL 2019 (30)	380	380	46.65 ± 7.43	45.01 ± 6.28	Alprazolam	Acupoint Application	1 week	Yongquan
Luo T 2019 (31)	31	31	NA	NA	Analgesics	Acupoint Application	1 weeks	Shenque, Yongquan, Zusanli
Lin JM 2022 (32)	30	30	NA	NA	Routine Treatmentt	Acupoint Application	7 weekss	Qimen
Mai YQ 2019 (33)	34	34	NA	NA	Doxepin Hydrochloride	Auricular Acupoints	4 weeks	Subcortical, Sympathetic, et al.
Wang HJ 2018 (34)	40	40	45.7	46.1	Fluoxetine Hydrochloride	Auricular Acupoints	4 weeks	Liver, Heart, Kidney, Portal, Subcortex
Shi YF 2021 (35)	50	50	48.21 ± 4.86	48.22 ± 5.02	Doxepin Hydrochloride	Auricular Acupoints	4 weeks	Henmen, Edge Middle, Under Cortex
Lv XA 2015 (36)	30	30	42 ± 5	43 ± 6	Flupentixol and Melitracen	Auricular Acupoints	4 weeks	Shenmen, Kidney, Liver, Heart, et al.
Bai T 2019 (37)	48	49	42.7 ± 3.02	43.1 ± 2.37	Oxycodone Hydrochloride	Auricular Acupoints	12 days	Shenmen, Lung, Pancreas, et al.
Han JF 2017 (38)	48	47	60.44 ± 8.29	56.51 ± 10.79	Flupentixol and Melitracen	Auricular Acupoints	6 weeks	Five Zang Organs, Ear Shenmen, Internal Secretion
Ge YF 2018 (39)	55	55	45.2 ± 3.7	45.8 ± 3.5	Doxepin Hydrochloride	Auricular Acupoints, Acupressure	4 weeks	Acupressure: Taiyang, Fengchi, et al.
Liu GL 2016 (40)	20	20	55.9 ± 2.4	56.2 ± 2.5	Alprazolam	Acupuncture, Moxibustion,Auricular Acupoints	5 weeks	Acupuncture and Moxibustion: Fengchi, et al.
Xiao B 2014 (41)	30	30	52 ± 5	51 ± 5	Fluoxetine Hydrochloride	Acupuncture,Auricular Acupoints	8 weeks	Acupuncture: Taichong, Hegu, et al.
Wang Y 2022 (42)	30	30	51.33 ± 8.64	54.93 ± 8.08	Paroxetine Hydrochloride	Acupuncture, Moxibustion	4 weeks	Ganshu, Xinshu, Pishu, Shenshu
Chen J 2018 (43)	18	18	66.43 ± 5.43	65.13 ± 5.98	Paroxetine Hydrochloride	Acupuncture, Moxibustion	6 weeks	Acupuncture: Neiguan, et al. Moxibustion: Guanmen, et al.

Exp, Experimental group; Con, Control group; NA, Not applicable.

### 3.3 Evaluation of literature quality and assessment of bias risk

The Kirkland Collaborative Network Bias Risk Assessment Tool was used to evaluate 30 included studies. The results showed that in terms of random sequence generation, 21 studies were at low risk, 8 studies were of unknown risk, and 1 study was at high risk; in terms of allocation concealment, 4 studies were at low risk, 25 studies were of unknown risk, and 1 study was at high risk; due to the operational characteristics of traditional Chinese acupuncture-related therapies, the blinding aspect of acupuncture and moxibustion treatment was only 1 study at low risk, and 29 studies were of unknown risk; all studies in the complete data and selective reporting of the results were judged to be at low risk, and the other bias risks were unknown (**Supplementary Figures 1, 2**).

Among the three main outcome indicators, the funnel plots of total response rate, SDS score and HAM-D score were not significantly asymmetrical, but some differences could still be seen. This may be due to factors such as differences in sample size of included studies, differences in treatment options, etc., rather than systematic publication bias (**Supplementary Figure 3**).

### 3.4 The total effective rate

**Figure 3:**
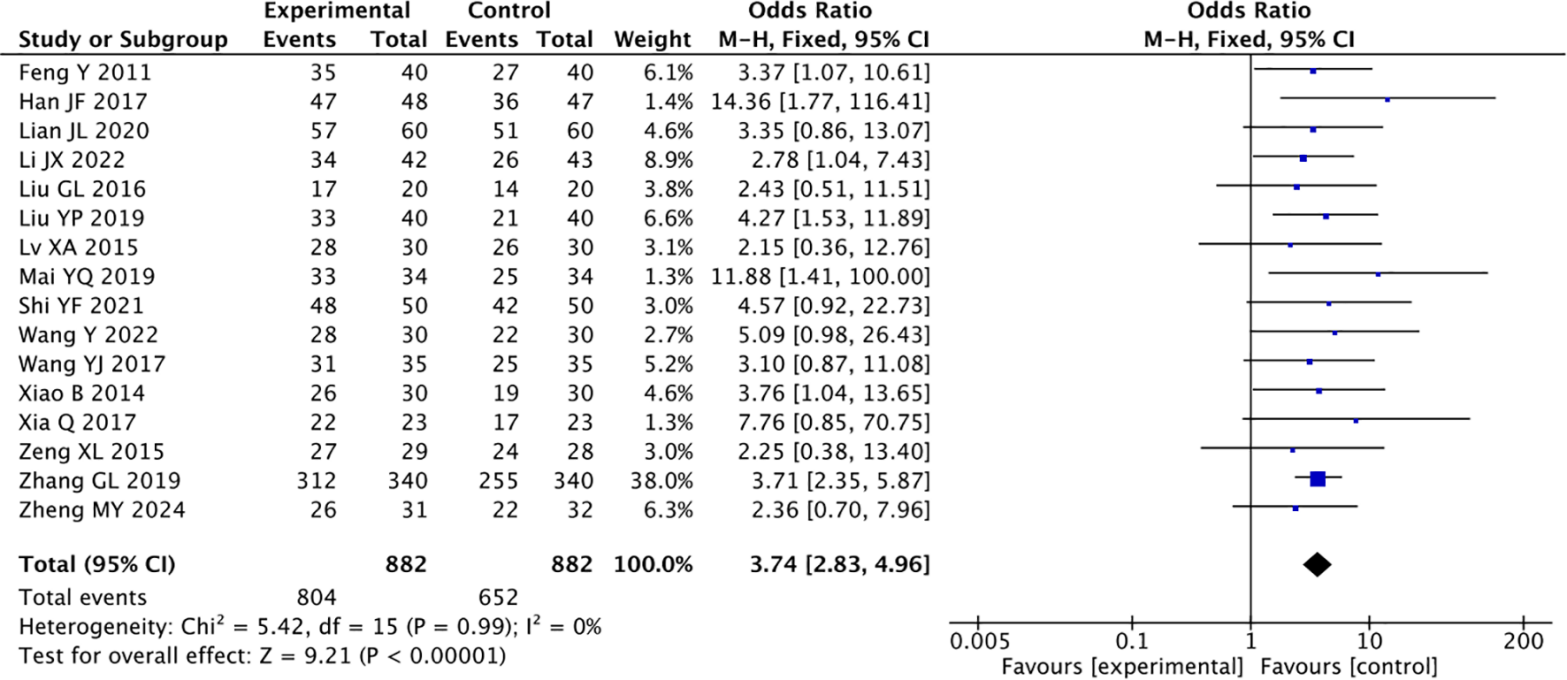
Forest plot of the overall effectiveness rate of combined traditional Chinese acupuncture-related therapies and drug therapy versus drug therapy alone.

Among the 16 references which reported the efficacy rate of traditional Chinese acupuncture-related therapies in treating CRD, the heterogeneity was low (*I^2^* = 0%), so a fixed-effects model was used. A total of 1764 patients were studied, including 882 patients in the experimental group, with an overall efficacy rate of 91.2% after the use of acupuncture-related therapies combined with psychotropic drugs; and 882 patients in the control group with an overall efficacy rate of 73.9% after the use of psychotropic drugs alone. At the same time, the results showed that acupuncture-related therapies was meaningful for CRD treatment (**Figure 3**).

Acupuncture combined with drug therapy was superior to drug therapy alone (OR = 3.28, *P* < 0.00001). The effective rate of the experimental group was 88.3%, and of the control group was 70.7%. Acupoint pressing and application combined with drug therapy were superior to drug therapy alone (OR = 3.64, *P* < 0.00001). The effective rate of the experimental group was 91.5%, and of the control group was 74.7%. Auricular acupressure combined with drug therapy was superior to drug therapy alone (OR = 6.32, *P* < 0.0001). The effective rate of the experimental group was 96.3%, and of the control group was 80.1%. The results showed that 2–3 kinds of traditional Chinese acupuncture-related therapies combined with drug therapy were better than drug therapy alone (OR = 4.25, *P* = 0.004). The effective rate of the experimental group was 91.8%, and of the control group was 72.6%(MD = 3.74, 95% CI 2.83, 4.96; *P* < 0.00001, **Table 2**).

**Table 2:** Subgroup analysis of the total effective rate after treatment based on the type of traditional Chinese acupuncture-related therapies.

Project	Test for heterogeneity		Analysis model	Test for overall effect		Mean difference 95% CI	Sample size	Effective rate (%)
	I^2^ (%)	*P*-Value		*Z*	*P*-Value		(Exp/Con)	
Acupuncture	0	0.97	Fixed	4.96	<0.00001	3.28 (2.05 to 5.25)	265/266	88.3
Acupoint pressing and Acupoint application	0	0.79	Fixed	5.88	<0.00001	3.64 (2.37 to 5.60)	375/375	91.5
Auricular acupoints	0	0.48	Fixed	4.03	<0.0001	6.32 (2.58 to 15.49)	162/161	96.3
Combination therapies	0	0.66	Fixed	2.87	0.004	0.19 (0.07 to 0.31)	73/73	91.8

Exp, Experimental group; Con, Control group; CI, Confidence interval.

### 3.5 Self-rating depression scale

**Figure 4:**
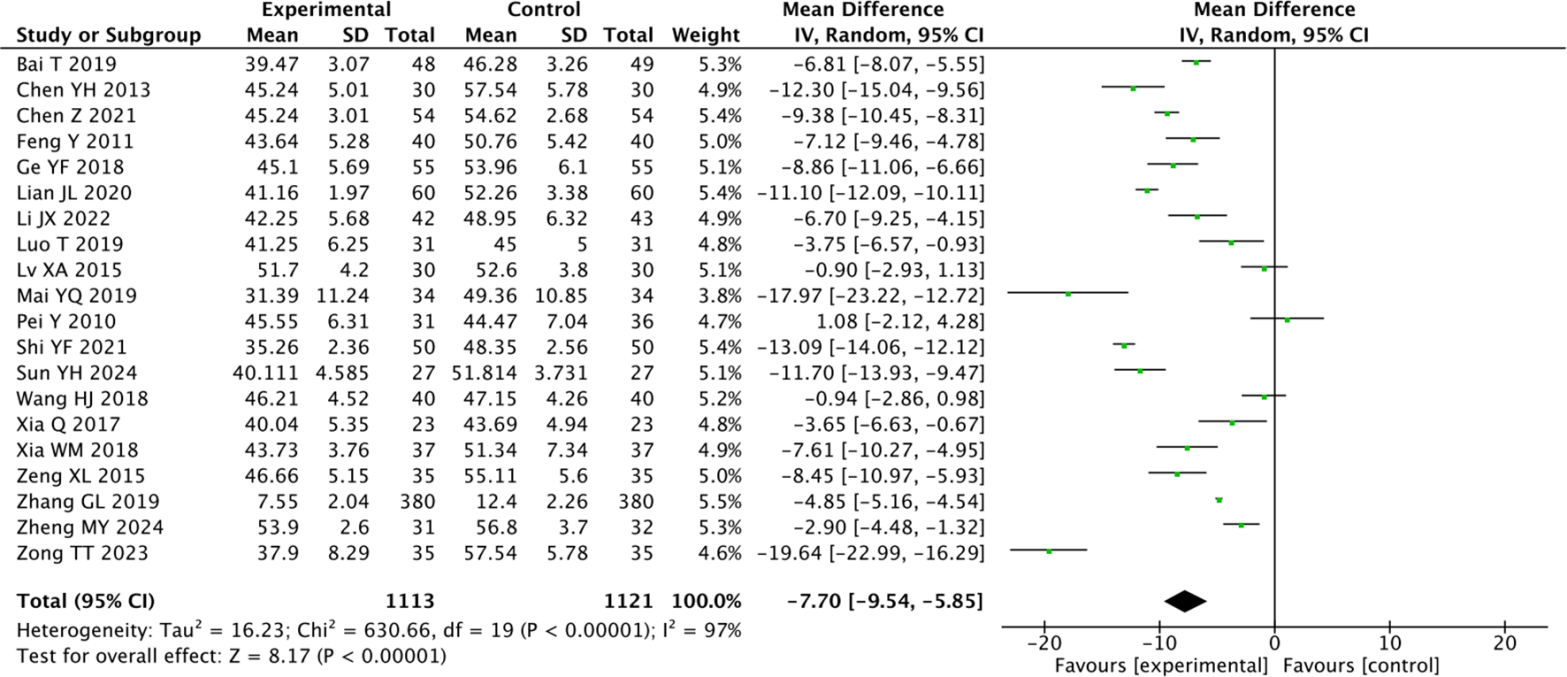
Forest plot of the Self-rating Depression Scale for combined traditional Chinese acupuncture-related therapies and drug therapy versus drug therapy alone.

Among the 20 articles that reported SDS scores, the heterogeneity was large ({it}I{sp}2{/it} {/sp}= 97%), so the random-effects model was used. A total of 2234 patients were enrolled, including 1113 patients in the experimental group and 1121 patients in the control group. The results showed that traditional Chinese acupuncture-related therapies could be used as an adjunct to drugs in the treatment of CRD (MD = -7.70, 95% CI -9.54, -5.85; *P* < 0.00001, **Figure 4**).

Acupuncture combined with medication was superior to medication alone (MD = -6.17, *P* < 0.00001). Moxibustion combined with medication was superior to medication alone (MD = -8.84, *P* < 0.00001). Acupoint application and pressing combined with drug therapy were superior to drug therapy alone (MD = -4.84, *P* < 0.00001). Auricular acupressure combined with drug therapy was superior to drug therapy alone (MD = -7.68, *P* = 0.006, **Table 3**).

**Table 3:** Subgroup analysis of SDS after treatment based on the type of traditional Chinese acupuncture-related therapies.

Project	Test for heterogeneity		Analysis model	Test for overall effect		Mean difference 95% CI	Sample size
	I^2^ (%)	*P*-Value		*Z*	*P*-Value		(Exp/Con)
Acupuncture	94	<0.00001	Random	4.56	<0.00001	-6.17 (-8.82 to -3.52)	316/323
Moxibustion	87	<0.0001	Random	4.65	<0.00001	-8.84 (-12.57 to -5.11)	129/129
Acupoint pressing and acupoint application	0	0.45	Random	31.15	<0.00001	-4.84 (-5.14 to -4.53)	411/411
Auricular acupoints	98	<0.00001	Random	2.75	0.006	-7.68 (-13.15 to -2.20)	202/203

Exp, Experimental group; Con, Control group; CI, Confidence interval.

### 3.6 Hamilton depression scale

**Figure 5:**
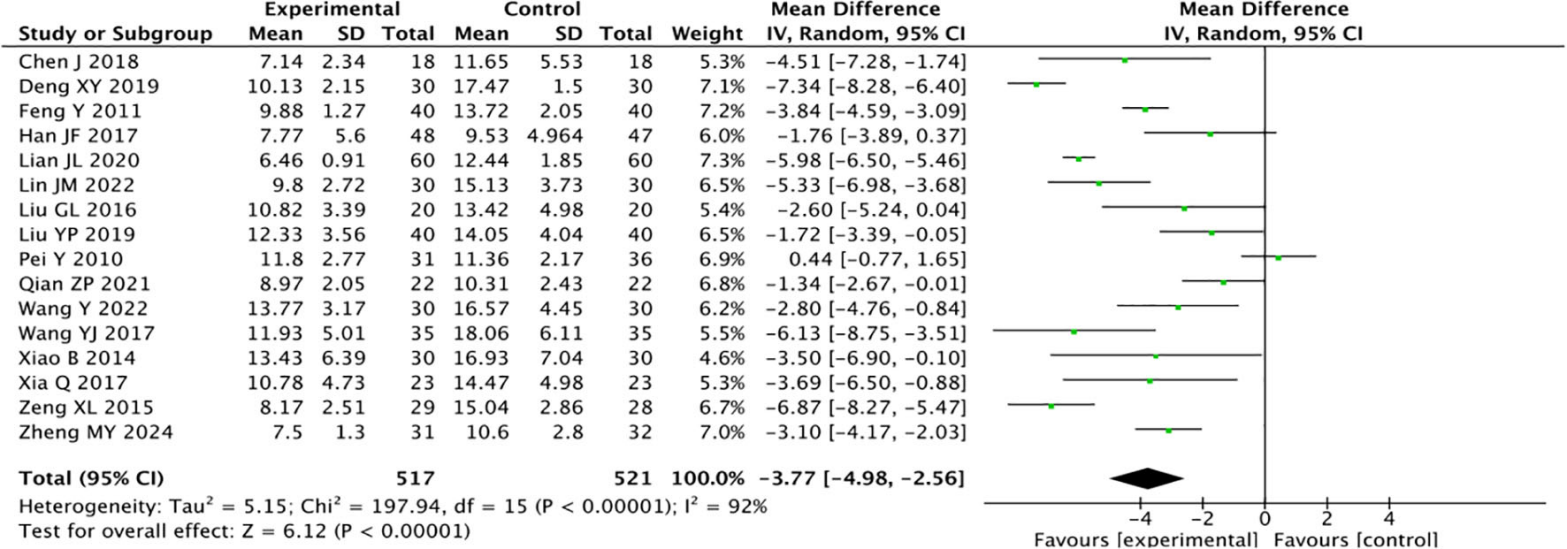
Forest plot of the Hamilton Depression Scale for combined traditional Chinese acupuncture-related therapies and drug therapy versus drug therapy alone.

Sixteen studies reported the HAM-D for traditional Chinese acupuncture-related therapies in the treatment of CRD. There was considerable heterogeneity among the studies ({it}I{sp}2{/it} {/sp}= 92%), so a random-effects model was adopted. A total of 1,038 patients were included: 517 in the experimental group and 521 in the control group. The results indicated that traditional Chinese acupuncture-related therapies could assist drug therapy for CRD and improve the psychological state of patients (MD = -3.77, 95% CI -4.98, -2.56; *P* < 0.00001, **Figure 5**).

Acupuncture combined with drug therapy was superior to drug therapy alone (MD = -4.05, *P* < 0.00001). Acupoint application and pressing combined with drug therapy were superior to drug therapy alone (MD = -5.56, *P* < 0.00001). Applying 2–3 kinds of traditional Chinese acupuncture-related therapies combined with drug therapy was better than drug therapy alone (MD = -3.21, *P* < 0.00001, **Table 4**).

**Table 4:** Subgroup analysis of HAM-D after treatment based on the type of traditional Chinese acupuncture-related therapies.

Project	Test for heterogeneity		Analysis model	Test for overall effect		Mean difference 95% CI	Sample size
	I^2^ (%)	*P*-Value		*Z*	*P*-Value		(Exp/Con)
Acupuncture	96	<0.00001	Random	4.63	<0.00001	-4.05 (-5.77 to -2.34)	284/289
Acupoint pressing and acupoint application	0	0.61	Random	7.80	<0.00001	-5.56 (-6.95 to - 4.16)	65/65
Combination therapies	0	0.74	Random	4.96	<0.00001	-3.21 (-4.48 to - 1.94)	98/98

Exp, Experimental group; Con, Control group; CI, Confidence interval.

### 3.7 Self-rating anxiety scale

**Figure 6:**
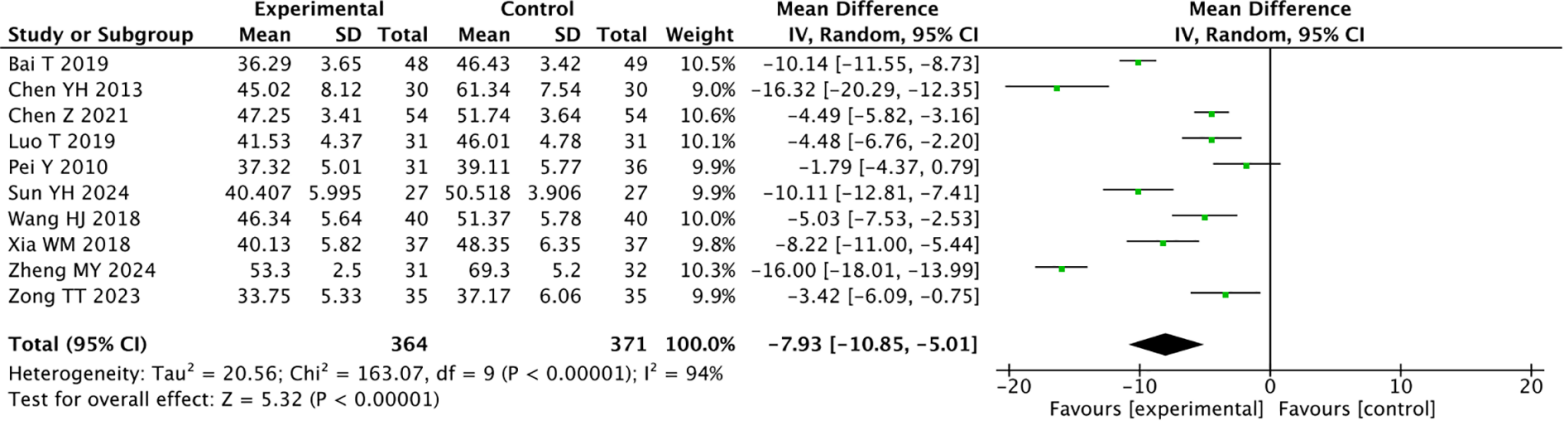
Forest plot of the Self-rating Anxiety Scale for combined traditional Chinese acupuncture-related therapies and drug therapy versus drug therapy alone.

The self-rating anxiety scale was reported in 10 articles. Since the heterogeneity among them was large ({it}I{sp}2{/it} {/sp}= 94%), the random-effects model was used. A total of 735 patients were enrolled: 364 in the experimental group and 371 in the control group. The results showed that acupuncture-related therapies could be used as an adjunct to medication in the treatment of CRD (MD = -7.93, 95% CI -10.85, -5.01; *P* < 0.00001, **Figure 6**).

### 3.8 Quality of life questionnaire: core 30 scale

The heterogeneity of the three main symptom domains was high, so the random-effects model was used. We found that traditional Chinese acupuncture-related therapies could significantly improve the efficacy of CRD in addition to drug therapy in the domains of pain (*P* = 0.01), nausea and vomiting (*P* = 0.004), fatigue (*P* = 0.0006), constipation (*P* = 0.01), diarrhea (*P* < 0.00001), dyspnea (*P* = 0.01), insomnia (*P* = 0.0002), and loss of appetite (*P* = 0.0009). In the five functional domains, the score heterogeneity was high so the random-effects model was used. There were significant differences in the domains role function (*P* = 0.03), body function (*P* < 0.0001), cognitive function (*P* = 0.0002), emotional function (*P* < 0.00001), and social function (*P* = 0.08, **Table 5**).

**Table 5:** Analysis of quality of life assessment scale and other outcome measures.

Project	Test for heterogeneity		Analysis model	Test for overall effect		Mean difference 95% CI	Sample size
	I^2^ (%)	*P*-Value		*Z*	*P*-Value		(Exp/Con)
1.Quality of life questionnaire-core 30 scale							
Main symptom domian score							
Pain	89	<0.00001	Random	2.52	0.01	-6.55 (-11.65 to -1.45)	196/195
Nausea and vomiting	96	<0.00001	Random	2.86	0.004	-4.67 (-7.87 to -1.47)	159/158
Fatigue	96	<0.00001	Random	3.41	0.0006	-9.14 (-14.40 to -3.89)	223/222
Single domain score							
Constipation	82	0.004	Random	2.49	0.01	-10.51 (-18.78 to -2.24)	119/118
diarrhea	75	0.02	Random	5.28	<0.00001	-11.08 (-15.20 to -6.97)	119/118
dyspnea	86	0.0006	Random	2.54	0.01	-9.80 (-17.36 to -2.24)	119/118
Insomnia	85	<0.0001	Random	3.72	0.0002	-9.53 (-14.55 to -4.52)	183/182
Loss of appetite	99	<0.00001	Random	3.31	0.0009	-14.93 (-23.77 to -6.09)	186/185
Economic hardship	97	<0.00001	Random	0.65	0.52	6.59 (-13.33 to 26.51)	89/88
Function domain score							
Role Function	94	<0.00001	Random	2.18	0.03	7.27 (0.73 to 13.82)	201/200
Body Function	85	<0.00001	Random	5.24	<0.0001	10.63 (6.65 to 14.60)	201/200
Cognitive Function	79	0.0002	Random	3.78	0.0002	7.20 (3.46 to 10.93)	238/237
Emotional Function	0	0.43	Fixed	14.26	<0.00001	9.47 (8.17 to 10.78)	238/237
Social Function	96	<0.00001	Random	1.74	0.08	6.23 (-0.79 to 13.25)	232/237
2.Other outcome measures							
PSQI	84	<0.0001	Random	6.40	<0.00001	-3.49 (-4.55 to -2.42)	560/559
NRS	92	<0.00001	Random	3.21	0.001	-1.34 (-2.16 to -0.52)	107/107
SF-36	88	0.003	Random	4.5	<0.00001	11.89 (6.71 to 17.07)	92/93

Exp, Experimental group; Con, Control group; CI, Confidence interval.

### 3.9 Other outcome measures

For the PSQI, there were 560 patients in the experimental group and 559 patients in the control group. The combination of acupuncture-related therapies and drug therapy was better than drug therapy alone (*P* < 0.00001). In the cancer pain NRS, there were 107 cases in each group. Traditional Chinese acupuncture-related therapies combined with drug therapy was better than drug therapy alone (*P* = 0.001). In the 36-Item Short Form Survey (SF-36), there were 92 cases in the experimental group and 93 cases in the control group. Traditional Chinese acupuncture-related therapies combined with drug therapy was better than drug therapy alone (*P* < 0.00001, **Table 5**).

## 4 Discussion

Depression is common among cancer patients, profoundly influencing their mental health and quality of life. Since the suicide rate is significantly elevated compared to the person without cancer, particularly in cancer types with poor prognosis, early intervention can improve survival rate. Western medical treatments for CRD mainly encompass psychiatric drugs and psychotherapy. Previous studies indicate that when patients with CRD use psychiatric drugs, various adverse reactions and side effects may occur, mainly including headache, nausea, and insomnia. These side effects can reduce the patients’ quality of life and affect their compliance with treatment (44–46).

The ongoing sedative drugs crisis in the United States has exacerbated the challenges surrounding cancer treatment, and as a result government organizations have called for the adoption of non-pharmaceutical intervention approaches (47). Research demonstrates that traditional Chinese acupuncture-related therapies, as a non-pharmaceutical intervention modality, has been widely employed for various diseases since it can reduce the side effects of drug treatment and strengthen the physical and mental well-being of patients. It has potential for assisting anti-tumor treatment and facilitating the overall rehabilitation of patients. Therefore, this study investigated the treatment of CRD using traditional Chinese acupuncture-related therapies.

At the initial stage of the research, we employed random effect models and fixed effect models for statistical analysis to mitigate the influence of these differences on the research results. Additionally, subgroup analyses were conducted to explore the impact of different intervention strategies on the outcome indicators, thereby providing more precise reference data for clinical applications. It should be noted that the significant differences in the research results may be attributed to the differences in pathological types, disease stages, and intervention methods (acupuncture, moxibustion, acupoint application, auricular point, auricular acupressure) and intervention degrees among the tumor patients included in the study. However, all the data were derived from standardized medical databases of large hospitals, and the included cases strictly followed unified diagnostic criteria, inclusion and exclusion criteria. Although there was a high degree of heterogeneity among the studies, all the included studies confirmed the significant clinical efficacy of acupuncture-related therapies in treating tumor-related depression, and this therapy has been clinically applied in multiple countries worldwide (48). Therefore, the research results have good internal validity and external applicability, providing evidence-based medical basis for the standardized implementation of acupuncture therapy in cancer adjunctive treatment. This is of great guiding significance for clinical practice.

The research findings indicated that, in contrast to drug therapy alone, traditional Chinese acupuncture-related therapies significantly improved depression, anxiety, insomnia and quality of life in patients with CRD. This observation is in accordance with previous studies and reviews (49, 50). Among the studies we included, the four most commonly-used acupoints were Baihui, Yintang, Neiguan, and Shenmen (**Supplementary Figure 4**). Acupuncture at Baihui can not only elevate Yang Qi to regulate and invigorate the mind but also pacify the liver and reduce internal heat to smooth emotions, enabling “the static to become dynamic” and improving depressive mood (51). The Yintang acupoint is located on the human head, belonging to the Governor Vessel Meridian and being an extraordinary acupoint outside the regular meridians. It governs all Yang Qi. Acupuncture at this point can regulate Yang Qi to subdue hyperactivity of Yang, open the orifices and restore consciousness (52). It is often combined with Baihui as an effective acupoint on the head for treating depression disorders (53). Neiguan is the connecting acupoint of the Pericardium Meridian, intersecting with the Yinwei Meridian and communicating with the Conception Vessel. Acupuncture at Neiguan can calm the mind and spirit, eliminate phlegm and dampness, and relieve depression and restlessness (54). Shenmen is the original acupoint of the Heart Meridian of Hand-Shaoyin, having the effect of tonifying Heart Qi and ameliorating depressive symptoms (55). These acupoints exhibit a remarkable ameliorative effect in the treatment of CRD (56).

In this study, the effective rate of the experimental group was 91.2%, while that of the control group was 73.9%. The effective rate of the experimental group was 17.3% higher than the control group. In the subgroup analysis of the overall effective rate, study results revealed that the effective rate of auricular acupressure in the experimental group was higher than combined therapy, acupoint sticking and pressing and acupuncture. Some studies have indicated that radiotherapy and chemotherapy are prone to enhance the neural sensitivity and pain perception of cancer patients (57). The auricular nerve is rich in distribution, including vagus nerve, trigeminal nerve and other branches, and auricular points are closely related to human viscera and meridians (58). Auricular pressure activates the vagus-cholinergic anti-inflammatory pathway by stimulating nerve endings, regulates the secretion of central neurotransmitters, increases the release of serotonin and dopamine, and reduces the excessive secretion of norepinephrine, thereby improving depressive mood (59). Compared with invasive therapies such as acupuncture and moxibustion, this method does not need to puncture the skin, and is better tolerated to cancer patients with sensitive skin and enhanced pain perception after radiotherapy and chemotherapy. It can avoid stress reactions related to invasive procedures, which may be its better curative effect important reason.

The results of this study show that there are differences in the SDS (MD = -7.70) and HAM-D (MD = -3.77) scores between the combined treatment group and the single medication group. According to the clinical research standards, a reduction of ≥5 points in the SDS score and ≥3 points in the HAM-D score is considered clinically significant (60). In this study, both score reductions in the combined treatment group met the standards, suggesting that this combined intervention plan has clear clinical value and significant effects in improving depressive symptoms. Previous studies suggest that acupuncture has high precision and can stimulate specific acupoints to produce corresponding therapeutic effects. Many patients can experience symptom relief immediately after receiving acupuncture treatment for the corresponding symptoms (61). Moxibustion combined with drug treatment was superior to drug treatment alone. One study (62) showed that the thermal effect produced by moxibustion can promote blood circulation and alleviate muscle tension and pain. It is also postulated in some studies that moxibustion can alleviate depressive symptoms through targeting neuronal and synaptic remodeling and immune responses (51). Acupoint pressing or acupoint massage combined with drug treatment was better than drug treatment alone. Acupoint application and acupoint pressing can have a sustained effect on the acupoints and prolong the therapeutic effect (63). Compared with oral medication, acupoint application reduces stimulation to the gastrointestinal tract (64). In a previous study (65), regular acupoint pressing is beneficial for preventing the occurrence of diseases and has good alleviating effects on symptoms such as headache, insomnia, and indigestion. Auricular acupressure combined with drug treatment was superior to drug treatment alone. Auricular acupressure is simple to operate and does not require complex equipment, making it suitable for rapid treatment of various diseases (66). The results of this research also indicate that the combination of 2–3 physical therapies with drug treatment outperforms drug treatment alone.

In this study, the combined effect of traditional Chinese acupuncture-related therapies and drug treatment was evaluated through the SDS cale and the HAM-D scale. It was discovered that combined therapy was conspicuously superior to drug therapy alone in the treatment of CRD. Particularly, the combination of acupuncture-related therapies like acupuncture, moxibustion, acupoint pressing, and auricular acupressure with drugs not only provided immediate symptom relief but also prolonged the therapeutic effect through a sustained action. These therapies are simple to operate, suitable for rapid treatment, and can strengthen the body’s resistance, offering an effective adjunctive therapeutic approach for cancer patients.

This study has the following limitations. Firstly, multiple acupuncture-related therapies were employed in this study so we conducted subgroup analyses to compare the differences between different physical therapies to determine the source of heterogeneity. Secondly, the direct comparative research evidence among different acupuncture therapies is relatively weak, resulting in insufficient accuracy of the relevant analysis conclusions and statistical test efficacy. This field still needs to conduct more rigorous direct comparison studies with proper designs. Thirdly, the sample sizes of some studies are small, leading to a relatively low level of evidence. However, further multi-center and large-sample studies are needed. Moreover, the results also statistically correlated the relevant acupoints, providing an important basis for clinical application.

## 5 Conclusions

Traditional Chinese acupuncture-related therapies is safe and effective for CRD, and can effectively alleviate the depressive symptoms of patients, reduce the use of drugs, and enhance patients’ quality of life. Particularly, auricular acupressure therapy demonstrates the best therapeutic effect. Hence, for patients with CRD, employing traditional Chinese acupuncture-related therapies to assist drug therapy for CRD is a rational choice. The findings of this research offer evidence for clinical application and provide a theoretical support for reducing the occurrence of drug abuse.

## Data Availability

The original contributions presented in the study are included in the article/**Supplementary Material**. Further inquiries can be directed to the corresponding authors.
