# Supplementary material for: Clinical efficacy study on the combined treatment of cancer-related depression with traditional Chinese acupuncture-related therapies and drugs
Source: Front Psychiatry. 2026 Jan 5;16:1717290. doi: 10.3389/fpsyt.2025.1717290 (PMC12812995; doi:10.3389/fpsyt.2025.1717290)
Supplement: Supplementary file 1 [file DataSheet1.docx]

**Appendix 1 search strategies**

Clinical efficacy study on the combined treatment of cancer-related depression with traditional Chinese acupuncture-related therapies and drugs

Ying Zhou^1†^, Feiqing Wang^1,2†^, Bo Yang^1†^, Xu Yang^1^, Xiaoxu Chen^1^, Bingbing Li^1^, Yanqing Liu^1^, Zhenhua Liu^3^, Yang Liu^1*^, Dongxin Tang^1*^, Yanju Li^4*^

^1^ Clinical Medical Research Center, The First Affiliated Hospital of Guizhou University of Traditional Chinese Medicine, Guiyang, Guizhou Province, China.

^2^ Academy of Medical Engineering and Translational Medicine, Tianjin University, Tianjin City, China.

^3^ Department of Hepatobiliary Surgery, Guizhou Provincial People's Hospital, Guiyang, Guizhou Province, China.

^4^ Department of Hematology Oncology, Affiliated Hospital of Guizhou Medical University, Guiyang, Guizhou Province, China.

† These authors contributed equally to this work.

^*^ Corresponding author: Yang Liu. Clinical Medical Research Center, The First Affiliated Hospital of Guizhou University of Traditional Chinese Medicine, No. 71 Bao Shan North Road, Yunyan District, Guiyang, Guizhou Province 550001, China. E-mail: ly7878@163.com.

^*^ Corresponding author: Dongxin Tang. Clinical Medical Research Center, The First Affiliated Hospital of Guizhou University of Traditional Chinese Medicine, No. 71 Bao Shan North Road, Yunyan District, Guiyang, Guizhou Province 550001, China. E-mail: tangdongxintcm@163.com.

^*^ Corresponding author: Yanju Li. Department of Hematology, Affiliated Hospital of Guizhou Medical University, No. 4 Bei Jing Road, Yunyan District, Guiyang, Guizhou Province 550004, China. E-mail: lyj8181@163.com.

**Appendix 1. The search strategy for including the trials in the PubMed database.**

#1 (((((((((((((((((Neoplasms[MeSH Terms]) OR (Tumors[Title/Abstract])) OR (Neoplasia[Title/Abstract])) OR (Neoplasias[Title/Abstract])) OR (Neoplasm[Title/Abstract])) OR (Tumor[Title/Abstract])) OR (Cancer[Title/Abstract])) OR (Cancers[Title/Abstract])) OR (Malignant Neoplasm[Title/Abstract])) OR (Malignancy[Title/Abstract])) OR (Malignancies[Title/Abstract])) OR (Malignant Neoplasms[Title/Abstract])) OR (Neoplasm, Malignant[Title/Abstract])) OR (Neoplasms, Malignant[Title/Abstract])) OR (Benign Neoplasms[Title/Abstract])) OR (Neoplasms, Benign[Title/Abstract])) OR (Neoplasm, Benign[Title/Abstract])) OR (Benign Neoplasm[Title/Abstract])

#2 (((((Depression[MeSH Terms]) OR (Depressive Symptoms[Title/Abstract])) OR (Depressive Symptom[Title/Abstract])) OR (Symptom, Depressive[Title/Abstract])) OR (Emotional Depression[Title/Abstract])) OR (Depression, Emotional[Title/Abstract])

#3 #1 AND #2

#4 (((((((((((Acupuncture[MeSH Terms]) OR(Pharmacopuncture[Title/Abstract])) OR (Acupuncture Treatment[Title/Abstract])) OR (Acupuncture Treatments[Title/Abstract])) OR (Treatment, Acupuncture[Title/Abstract])) OR (Therapy, Acupuncture[Title/Abstract])) OR (Pharmacoacupuncture Treatment[Title/Abstract])) OR (Treatment, Pharmacoacupuncture[Title/Abstract])) OR (Pharmacoacupuncture Therapy[Title/Abstract])) OR (Therapy, Pharmacoacupuncture[Title/Abstract])) OR (Acupotomy[Title/Abstract])) OR

(Acupotomies[Title/Abstract])

#5 (Moxibustion[MeSH Terms]) OR (Moxabustion[Title/Abstract])

#6 ((((((((Massage[MeSH Terms]) OR (Zone Therapy[Title/Abstract])) OR (Therapies, Zone[Title/Abstract])) OR (Zone Therapies[Title/Abstract])) OR (Therapy, Zone[Title/Abstract])) OR (Massage Therapy[Title/Abstract])) OR (Massage Therapies[Title/Abstract])) OR (Therapies, Massage[Title/Abstract])) OR (Therapy, Massage[Title/Abstract])

#7 (((((((Acupuncture, Ear[MeSH Terms]) OR (Acupunctures, Ear[MeSH Terms])) OR (Ear Acupunctures[MeSH Terms])) OR (Acupuncture, Auricular[MeSH Terms])) OR (Acupunctures, Auricular[MeSH Terms])) OR (Auricular Acupunctures[MeSH Terms])) OR (Auricular Acupuncture[MeSH Terms])) OR (Ear Acupuncture[MeSH Terms])

#8 #4 OR #5 OR #6 OR #7

#9 ((Randomized Controlled Trial[Title/Abstract]) OR (RCT[Title/Abstract])) OR (random[Title/Abstract])

#10 #3 AND #8 AND #9

**Appendix 1. The search strategy for including the trials in the Cochrane Library.**

#1 MeSH descriptor: [Neoplasms] explode all trees

#2 (Neoplasm OR Benign OR Benign Neoplasm OR Benign Neoplasms OR Neoplasms, Benign OR Neoplasias OR Neoplasm OR Tumor OR Tumors OR Neoplasia OR Cancers OR Malignancy OR Cancer OR Neoplasm, Malignant OR Malignant Neoplasms OR Malignancies OR Neoplasms, MalignantOR Malignant Neoplasm):ti,ab,kw

#3 #1 OR #2

#4 MeSH descriptor: [Depression] explode all trees

#5 (Emotional Depression OR Depressive Symptoms OR Depressive Symptom OR Symptom, Depressive OR Depression, Emotional):ti,ab,kw

#6 #4 OR #5

#7 #3 AND #6

#8 MeSH descriptor: [Acupuncture] explode all trees

#9 (Pharmacopuncture):ti,ab,kw

#10 #8 OR #9

#11 MeSH descriptor: [Moxibustion] explode all trees

#12 (Moxabustion):ti,ab,kw

#13 #11 OR #12

#14 MeSH descriptor: [Massage] explode all trees

#15 (Therapies, Zone OR Therapy, Zone OR Zone Therapies OR Zone Therapy OR Massage Therapies OR Therapies, Massage OR Massage Therapy OR Therapy, Massage):ti,ab,kw

#16 #14 OR #15

#17 MeSH descriptor: [Acupuncture, Ear] explode all trees

#18 (Acupunctures, Auricular OR Acupuncture, Auricular OR Auricular Acupuncture OR Auricular Acupunctures OR Ear Acupuncture OR Ear Acupunctures OR Acupunctures, Ear):ti,ab,kw

#19 #17 OR #18

#20 #10 OR #13 OR #16 OR #19

#21 #3 AND #20

**Appendix 1. The search strategy for including the trials in the** **Web of Science.**

#1 (((((((((((((((((TS=(Neoplasms)) OR TS=(Tumors)) OR TS=(Neoplasia)) OR TS=(Neoplasias)) OR TS=(Neoplasm)) OR TS=(Tumor)) OR TS=(Cancer)) OR TS=(Cancers)) OR TS=(Malignant Neoplasm)) OR TS=(Malignancy)) OR TS=(Malignancies)) OR TS=(Malignant Neoplasms)) OR TS=(Neoplasm, Malignant)) OR TS=(Neoplasms, Malignant)) OR TS=(Benign Neoplasms)) OR TS=(Neoplasms, Benign)) OR TS=(Neoplasm, Benign)) OR TS=(Benign Neoplasm)

#2 (((((TS=(Depression)) OR TS=(Depressive Symptoms)) OR TS=(Depressive Symptom)) OR TS=(Symptom, Depressive)) OR TS=(Emotional Depression)) OR TS=(Depression, Emotional)

#3 #1 AND #2

#4 (((((((((((TS=(Acupuncture)) OR TS=(Pharmacopuncture)) OR TS=(Acupuncture Treatment)) OR TS=(Acupuncture Treatments)) OR TS=(Treatment, Acupuncture)) OR TS=(Therapy, Acupuncture)) OR TS=(Pharmacoacupuncture Treatment)) OR TS=(Treatment, Pharmacoacupuncture)) OR TS=(Pharmacoacupuncture Therapy)) OR TS=(Therapy, Pharmacoacupuncture)) OR TS=(Acupotomy)) OR TS=(Acupotomies)

#5 (TS=(Moxibustion)) OR TS=(Moxabustion)

#6 ((((((((TS=(Massage)) OR TS=(Zone Therapy)) OR TS=(Therapies, Zone)) OR TS=(Zone Therapies)) OR TS=(Therapy, Zone)) OR TS=(Massage Therapy)) OR TS=(Massage Therapies)) OR TS=(Therapies, Massage)) OR TS=(Therapy, Massage)

#7 (((((((TS=(Acupuncture, Ear)) OR TS=(Acupunctures, Ear)) OR TS=(Ear Acupunctures)) OR TS=(Acupuncture, Auricular)) OR TS=(Acupunctures, Auricular)) OR TS=(Auricular Acupunctures)) OR TS=(Auricular Acupuncture)) OR TS=(Ear Acupuncture)

#8 #4 OR #5 OR #6 OR #7

#9 ((TS=(Randomized Controlled Trial)) OR (TS=(RCT)) OR TS=(random)

#10 #3 AND #8 AND #10

**Appendix 1. The search strategy for including the trials in the** **Chinese Scientific Journal Database (VIP)**

题名或关键词: (癌症 or 癌 or 肿瘤 or 瘤) and题名或关键词: (抑郁 or 抑郁症or 情绪低落) and题名或关键词: (针 or 针灸 or 针刺 or 电针 or 头针 or 体针 or火针 or 温针灸or 艾灸or 灸or 灸法 or 耳穴 or 耳针 or 穴位注射 or 穴位敷贴 or 穴位)

**Appendix 1. The search strategy for including the trials in the Wanfang Data**

题名或关键词: (癌症 or 癌 or 肿瘤 or 瘤) and题名或关键词: (抑郁 or 抑郁症or 情绪低落) and题名或关键词: (针 or 针灸 or 针刺 or 电针 or 头针 or 体针 or火针 or 温针灸or 艾灸or 灸or 灸法 or 耳穴 or 耳针 or 穴位注射 or 穴位敷贴 or 穴位)

**Appendix 1. The search strategies for the included RCTs for** **China National Knowledge Infrastructure (CNKI)**

SU%='癌症'+'癌'+'肿瘤'+'瘤' AND SU%='抑郁'+'抑郁症'+'情绪低落' AND SU%='针'+'针灸'+'针刺'+'电针'+'头针'+'体针'+'火针'+'温针灸'+'艾灸'+'灸'+'灸法'+'耳穴'+'耳针'+'穴位注射'+'穴位敷贴'+'穴位'
