# Supplementary material for: Clinical efficacy study on the combined treatment of cancer-related depression with traditional Chinese acupuncture-related therapies and drugs
Source: Front Psychiatry. 2026 Jan 5;16:1717290. doi: 10.3389/fpsyt.2025.1717290 (PMC12812995; doi:10.3389/fpsyt.2025.1717290)
Supplement: Supplementary file 2 [file DataSheet2.docx]

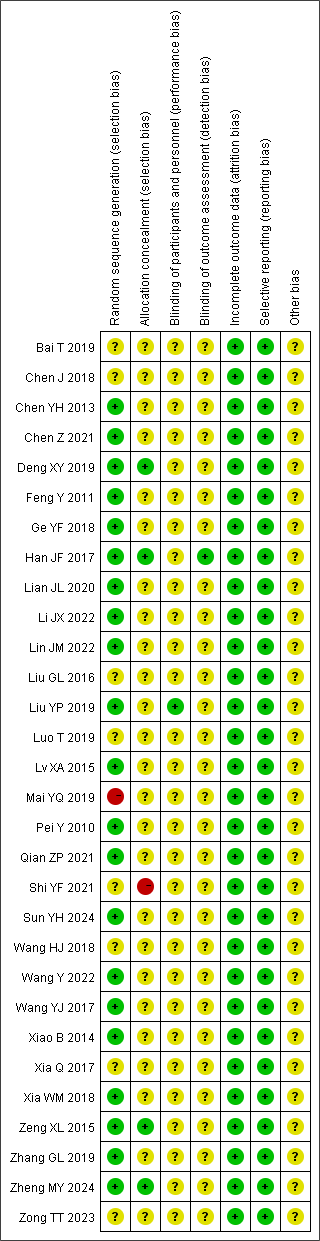


Supplement Figure 1: Risk of bias summary.


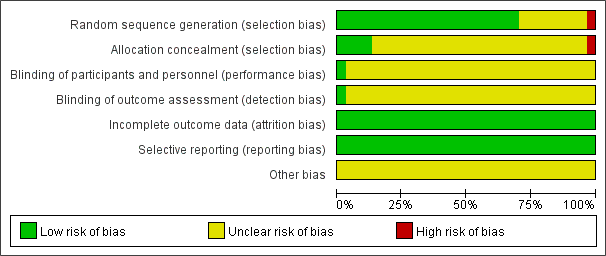
 Supplement Figure 2**:** Risk of bias graph.


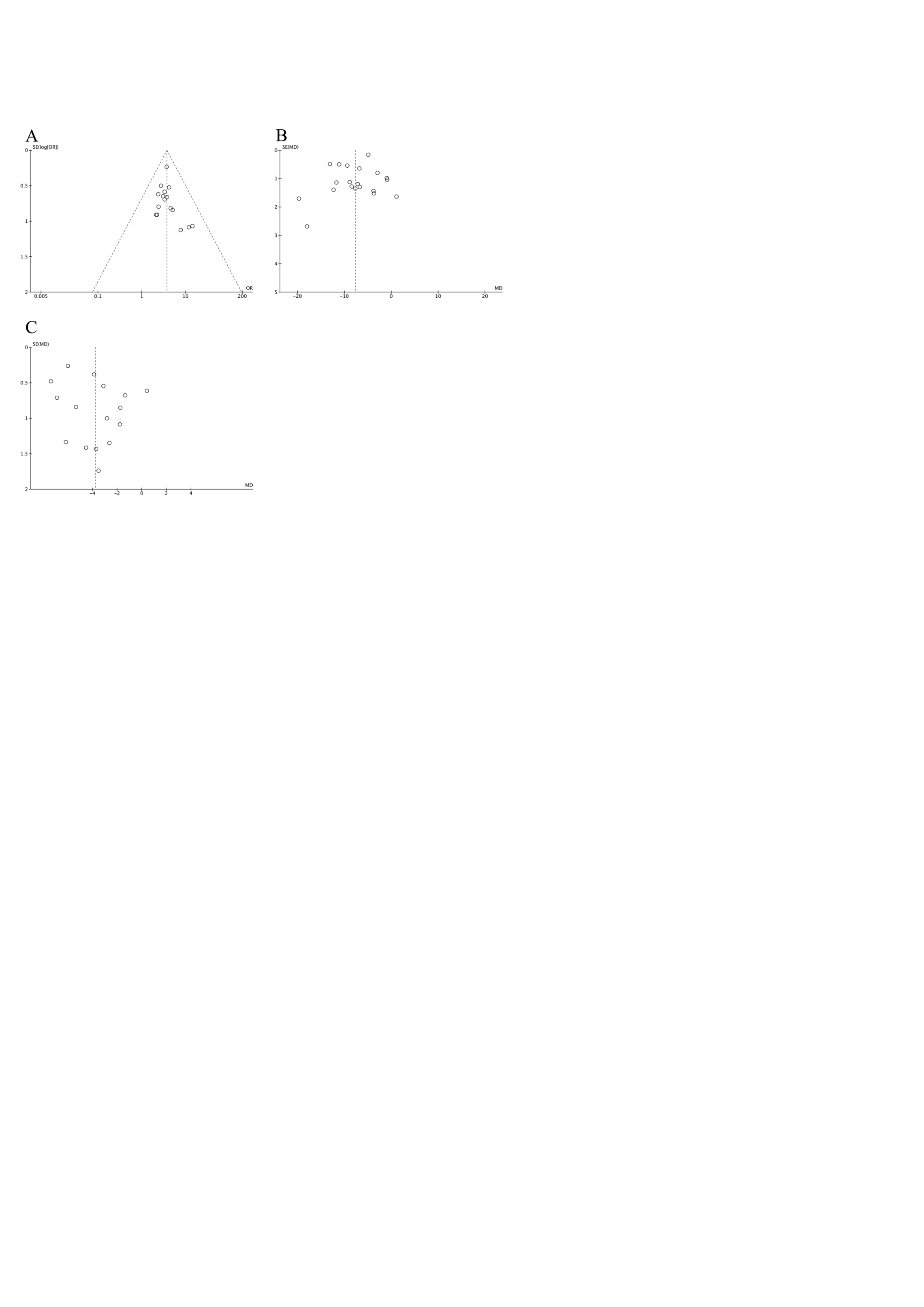


Supplement Figure 3**:** Funnel plot analysis to evaluate the results of publication bias. A: The funnel chart of total effective rate; B:The funnel chart of Self-rating depression scale; C: The funnel chart of Hamilton Depression Scale


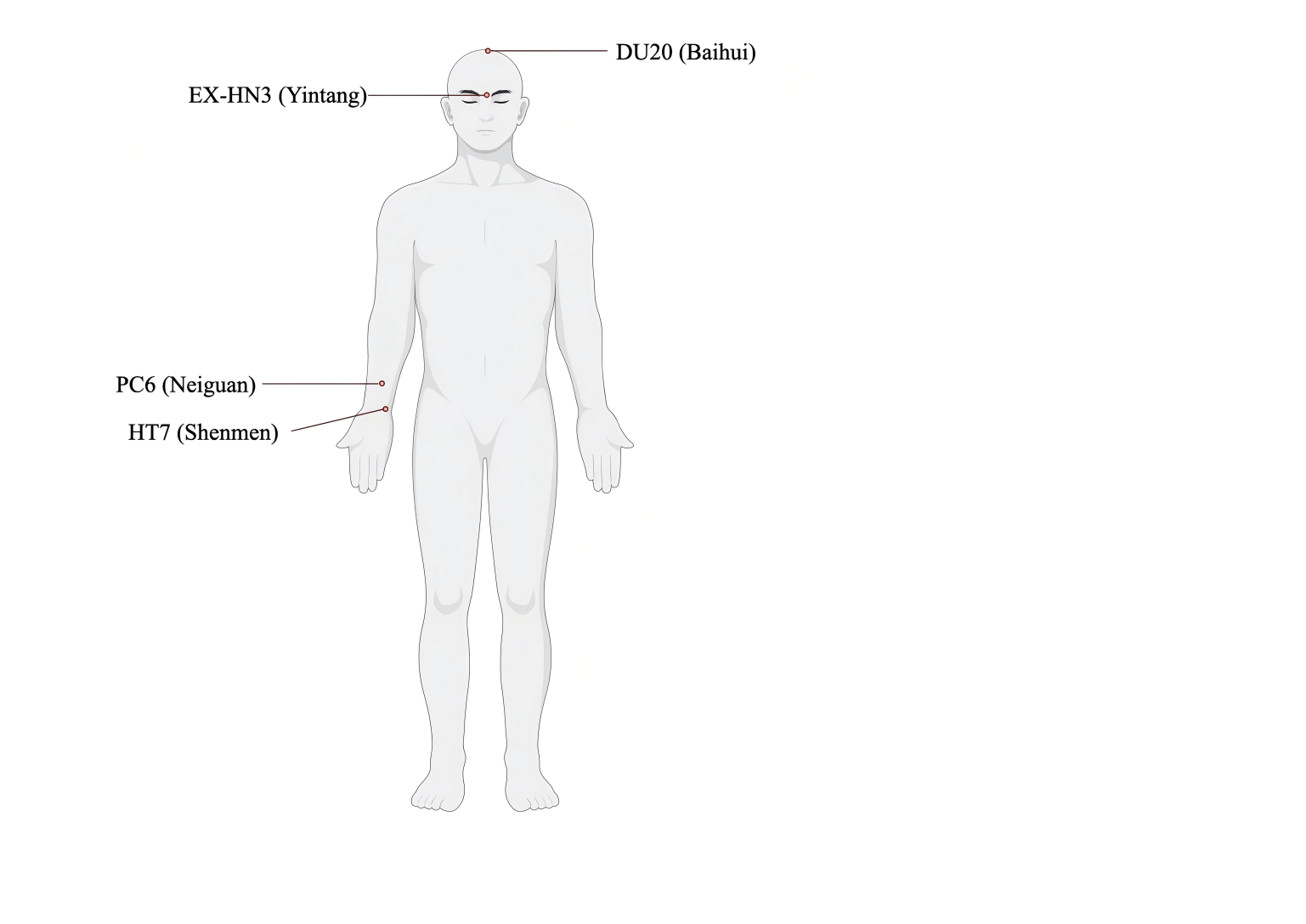


Supplement Figure 4**:** Acupoints were Baihui, Yintang, Neiguan, and Shenmen.
